# Quarantine

**Published:** 1878-10

**Authors:** William Bailey

**Affiliations:** Prof. Practice of Medicine, Hospital Medical College


### Quarantine.

By William Bailey, M. D., Prof. Practice of Medicine, Hospital Medical College.
Read before the Medico-Chirurgical Society.

The question of pestilential diseases is one of paramount importance, and its consideration in view of our relations to the South is now exceedingly apropos. It is not my desire to occupy the time of the society with unprofitable disputation. I wish to offer a few thoughts upon subjects out of which is being developed a policy ruinous to our Southern neighbors, both as to life and commerce.

Quarantine should never be established unless it be to protect us from diseases unquestionably contagious, and even then it is of doubtful propriety; otherwise it is a disgrace to the people adopting it. However, when quarantine is proper, then it may be held responsible for all legitimate deductions therefrom. If it is established, it ought to be of the strictest sort, else it is of no avail. Let us illustrate some of its effects: St. Louis, Cincinnati, and Louisville establish a strict quarantine as against New Orleans. Now, admitting this to be proper under the first law of nature, it holds good with additional force that Cairo, Memphis, and Vicksburg shall do the same thing. If these, then the smaller cities and towns adjacent shall protect themselves, till the parishes immediately around may prohibit any intercourse with the infected city. The consequence is legitimate that the citizens of New Orleans must be kept within its corporate limits, and it may be that the denizens of its purlieus of filth shall be compelled to remain in their haunts anticipating even the tortures of the condemned.

The consequences of legitimate quarantine when thus strictly enforced become repulsive to our better natures, tending to degrade our philanthropy by causing us to ignore those obligations that are imposed by the brotherhood of man. However, I shall not argue against quarantine from these considerations alone, but likewise from my convictions that none of these so-called pestilential diseases are in the slightest degree contagious. In the list of these are included cholera, plague, and yellow fever. Only in the last century England embraced in the list against which quarantine was to be established, in addition to these, typhus, jail, hospital, and ship fevers, dysentery, and even scurvy. This day places restrictions upon the first three alone.

It may be interesting to inquire into the history of quarantine. You will at once admit that it must result of necessity from convictions of the contagious nature of the affection. The curious student of medical literature will be interested to know that silence prevails as regards the contagious nature of these pestilential diseases till the year 1547, when it was advanced by Fracastorious in order to accomplish a religioso political object, namely, the removal of the Ecumenical Council of Trent to Bologna. This becomes from this date the doctrine of the Christian nations, as none dare oppose himself to the authority of it. From this teaching has sprung the practice of quarantine. We might argue against quarantine because it practically fails to accomplish its object; but if it can be shown that these diseases are not contagious, the necessity for it is removed. We desire to present some general principles alike applicable to all these diseases:

They are all of climatic origin, the specific cause in each case operating through or by means of the atmosphere.

No disease limited by latitude, longitude, or altitude is contagious. No disease, in one instance propagated by contagion, is ever produced in any other way. The opposite of this is likewise true. No disease produced at one time by any other means than by contagion is ever produced by contact.

These diseases are limited to certain districts, never exceeding them, so of necessity there must exist within those districts, and in them alone, the causes of them. They prevail only in certain seasons of the year; so only in certain seasons of the year are the causes of them produced. They are often confined by certain definite lines, and even in cities they may be confined to certain parts of them, and to certain squares, streets, or even houses. They obey all the laws of endemic diseases, and not those of contagious affections. One of the greatest difficulties in the way of investigation in regard to their causation is the fact that the minds of men are already occupied by contagion, and hence this must be maintained, no matter what else fails. I will give you one illustration of this: A committee of physicians of Moscow in 1771 invented a fumigatory powder as a preventive against the plague. Clothing from pestiferous patients, saturated with sweat, pus, and icherous matter from pestiferous dead bodies were prepared with this powder and worn by twenty convicts for the space of one month without in a single instance causing the disease. The committee, of course, were delighted with their preventive, and a thousand such instances would never have caused even a shadow of a doubt to enter their heads as to infection in the clothing.

If you please, we will now be somewhat more specific, and consider some matters with regard to yellow fever.

So many facts in connection with the history of this disease precludes the possibility of contagion that I am at a loss to know how intelligent men can consider them and remain contagionists. It prevails only in certain latitudes, and by preference in isolated districts where sanitary measures are ignored or neglected. The cause seems to be more operative in the night than in the day time. The cause may be retained in the system for an indefinite time in a latent form, the disease being developed long after the person has been removed from the district it which it was contracted or received. Thousands of cases go to other parts of our country and do not prove nucleus for new development. The endemics as such are invariably cut short by a severe frost; the cases that occur in the district afterward are those who had received the cause before the frost, for new-comers are not affected.

You will readily appreciate from these statements why I am so bitterly opposed to measures of quarantine for the prevention of the spread of disease. Unless you exclude the well as well as the sick there can be no certainty of prevention, for some one may come into your midst in apparent good health, or even submit to quarantine or forty days detention, and then have developed in them the disease. No one could by any possibility detect such a case before its development. We find the world exercised to ascertain if by possibility an infected vessel has arrived upon our shores, not hesitating to believe that the epidemic has been developed from it, and yet not being attracted by the fact that upon a change of temperature the disease suddenly ceases, although fifty thousand cases may exist at the time. All London is agog to account for the introduction of cholera, and finally concludes that the drinking-water is possibly contaminated by the discharges from the bowels of two men sick with the disease, and yet no notice is taken of the fact that in a few weeks the epidemic very suddenly disappears while twenty thousand cases are adding their quantum to further contamination. We find the pestilence that devastated London in 1665, the year preceding the great fire, disappeared in the same way. At that time it was the custom to designate the houses in which the plague existed as we do now for variola, only that there was placed on the door a large red cross with the inscription beneath it "Lord have mercy upon us." I suppose they thought this more absolutely essential, inasmuch as they had none one for the other.

I am cognizant that I have presented this subject very imperfectly, but I feel that the question of contagion is the taproot upon which quarantine depends, and believing very fully that contagionists fail utterly to establish their doctrine, I oppose vehemently all such measures of prevention as quarantine.

If it was proper to discuss at any length in this paper the etiology and prophylaxis of these pestilential diseases, I should unhesitatingly say that they obey all the laws of so-called malarial affections. I do not insist that the essential cause is identical with that that produces an ordinary intermittent, but I do mean to say that in my opinion they are all dependent upon vegetable decomposition, moisture, and solar heat, and it is the variation in these three that causes the differences in the manifestations of the malarial affections. Accordingly, as we may vary these factors so will we have an ordinary intermittent, remittent, pernicious fever, cholera, yellow fever, or plague.

Having made this statement of my convictions, you will readily perceive that I think it about as sensible to establish quarantine against intermittent as yellow fever. I believe they are very largely subject to state medicine, and the best thing we can do for our neighbors is to advise them to cease reliance on such questionable measures as quarantine and substitution of one stink for another one, and to rely on sanitary and hygienic measures alone. Cleanliness is healthy as well as being akin to godliness. New Orleans needs again such an administration of its sanitary police affairs as was afforded by General Butler. Whenever either of the factors for production enumerated above is absent, then we can not by any possibility have yellow fever. It is for this reason that cities located upon or near the northern border limits of the yellow-fever zone are scourged only occasionally, and those situated differently have annual visitations. Now, we have not the power to control the factor of solar heat, but we can largely interfere with the concurrence of the events necessary to the production of the poison by cleanliness. With these views all possible circumstances in pestilential history are explained, but under the view of contagion confusion is worse confounded. "If facts do not support their theory, then so much the worse for the facts." If possible remove the sick, but at any rate the well, to a climate in which the disease is not generated. It may possibly be within a few miles or even rods of the seat of pestilence.

All hail to the magnanimous heroism of Holly Springs, the only city in my knowledge that has had enough of the milk of human kindness to do as she would be done by.—*Louisville News.*

